# Crystal structure characterization, Hirshfeld surface analysis, and DFT calculation studies of 1-(6-amino-5-nitro­naphthalen-2-yl)ethanone

**DOI:** 10.1107/S2056989024003797

**Published:** 2024-05-03

**Authors:** Xin-Wei Shi, Ming-Sheng Bai, Shao-Jun Zheng, Qiang-Qiang Lu, Gen Li, Ya-Fu Zhou

**Affiliations:** a Shaanxi Engineering Research Centre for Conservation and Utilization of Botanical Resources, Xi’an Botanical Garden of Shaanxi Province (Institute of Botany of Shaanxi Province), Xi’an 710061, People’s Republic of China; b School of Life Sciences, Ningxia University, Yinchuan 750021, People’s Republic of China; c School of Environmental and Chemical Engineering, Jiangsu University of Science and Technology, Zhenjiang 212003, People’s Republic of China; Venezuelan Institute of Scientific Research, Venezuela

**Keywords:** crystal structure, naphthalene ring, Hirshfeld surface analysis, DFT calculations

## Abstract

The title compound, C_12_H_10_N_2_O_3_, was obtained by the de­acetyl­ation reaction of 1-(6-amino-5-nitro­naphthalen-2-yl)ethanone in a concentrated sulfuric acid methanol solution. The mol­ecule comprises a naphthalene ring system bearing an acetyl group (C-3), an amino group (C-7), and a nitro group (C-8). In the crystal, the mol­ecules are assembled into a two-dimensional network by N⋯H/H⋯N and O⋯H/H⋯O hydrogen-bonding inter­actions. *n*–π and π–π stacking inter­actions are the dominant inter­actions in the three-dimensional crystal packing. Hirshfeld surface analysis indicates that the most important contributions are from O⋯H/H⋯O (34.9%), H⋯H (33.7%), and C⋯H/H⋯C (11.0%) contacts. The energies of the frontier mol­ecular orbitals were computed using density functional theory (DFT) calculations at the B3LYP-D3BJ/def2-TZVP level of theory and the LUMO–HOMO energy gap of the mol­ecule is 3.765 eV.

## Abstract 2

The title compound, C_12_H_10_N_2_O_3_, was obtained by the de­acetyl­ation reaction of 1-(6-amino-5-nitro­naphthalen-2-yl)ethanone in a concentrated sulfuric acid methanol solution. The mol­ecule comprises a naphthalene ring system bearing an acetyl, amino and nitro groups. In the crystal, the mol­ecules are assembled into a two-dimensional network by N⋯H/H⋯N and O⋯H/H⋯O hydrogen-bonding inter­actions. *n*–π and π–π stacking inter­actions are the dominant inter­actions in the three-dimensional crystal packing.

## 1. Chemical context

2-Naphthyl­amine (also known as *β*-naphthyl­amine, CAS 91-59-8) occurs as pink crystals under the influence of light and has a weak, aromatic odor. In the past, It has been used for ligands or surfactants for the production of azo dyes, as an anti­oxidant in the rubber industry, as well as in the cable industry (Czubacka *et al.*, 2020 ▸). It is also used for oxytocinase assays, water analysis, and sewage control, and as a model bladder carcinogen in laboratories (Freudenthal *et al.*, 1999 ▸). It is not currently produced on an industrial scale and is not found in the natural state. 
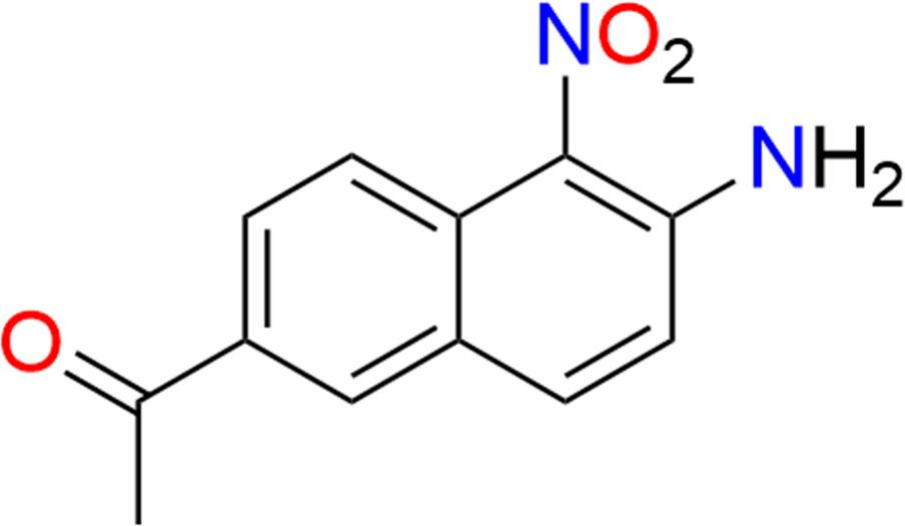


2-Naphthyl­amine derivatives find applications in organic synthesis and serve as building blocks in the synthesis of dyes (Czubacka *et al.*, 2020 ▸), pharmaceuticals (Wu *et al.*, 2024 ▸), and other organic compounds (Ding *et al.*, 2005 ▸; Yao *et al.*, 2013 ▸). The title 2-naphthyl­amine derivative, (**I**) was obtained by the de­acetyl­ation reaction of 2-acetyl-6-acetyl­amino-5-nitro­naphthalene in concentrated sulfuric acid methanol solution. Herein we report the crystal structure, Hirshfeld surface analysis, and density functional theory (DFT) calculations of the mol­ecule.

## 2. Structural commentary

**Figure 1:**
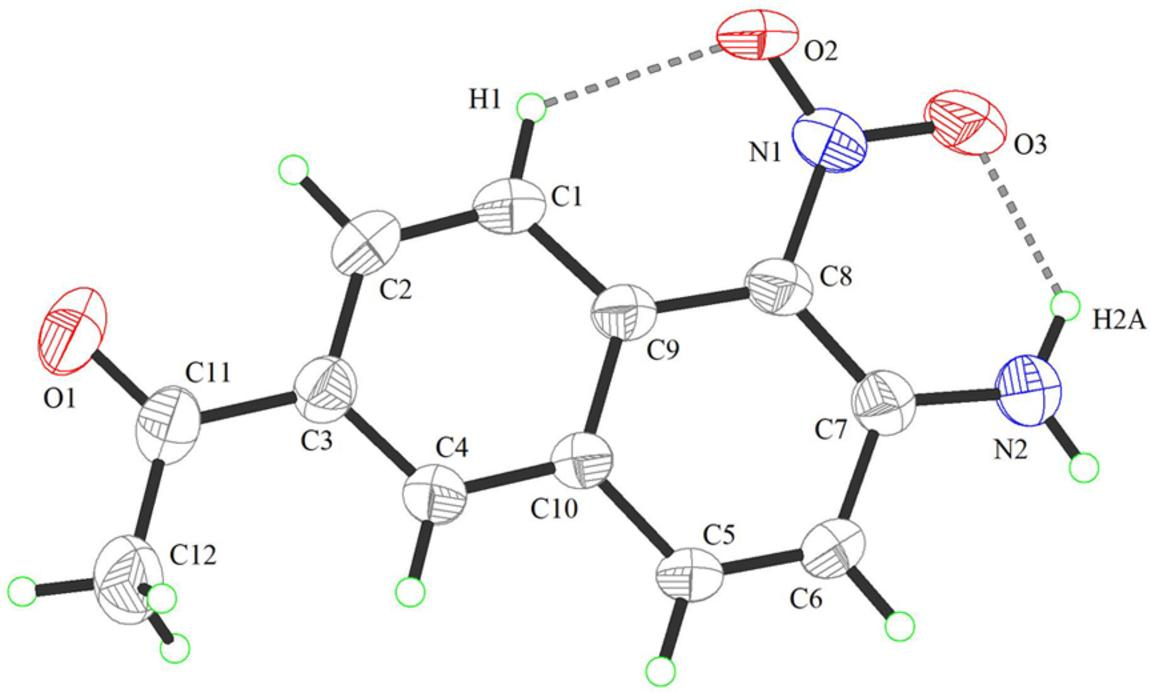
The title mol­ecule atomic numbering scheme. Displacement ellipsoids are depicted at the 50% probability level The C1—H1⋯O2 and N2—H2*A*⋯O3 intra­molecular hydrogen bonds are depicted by gray dashed lines.

**Figure 2:**
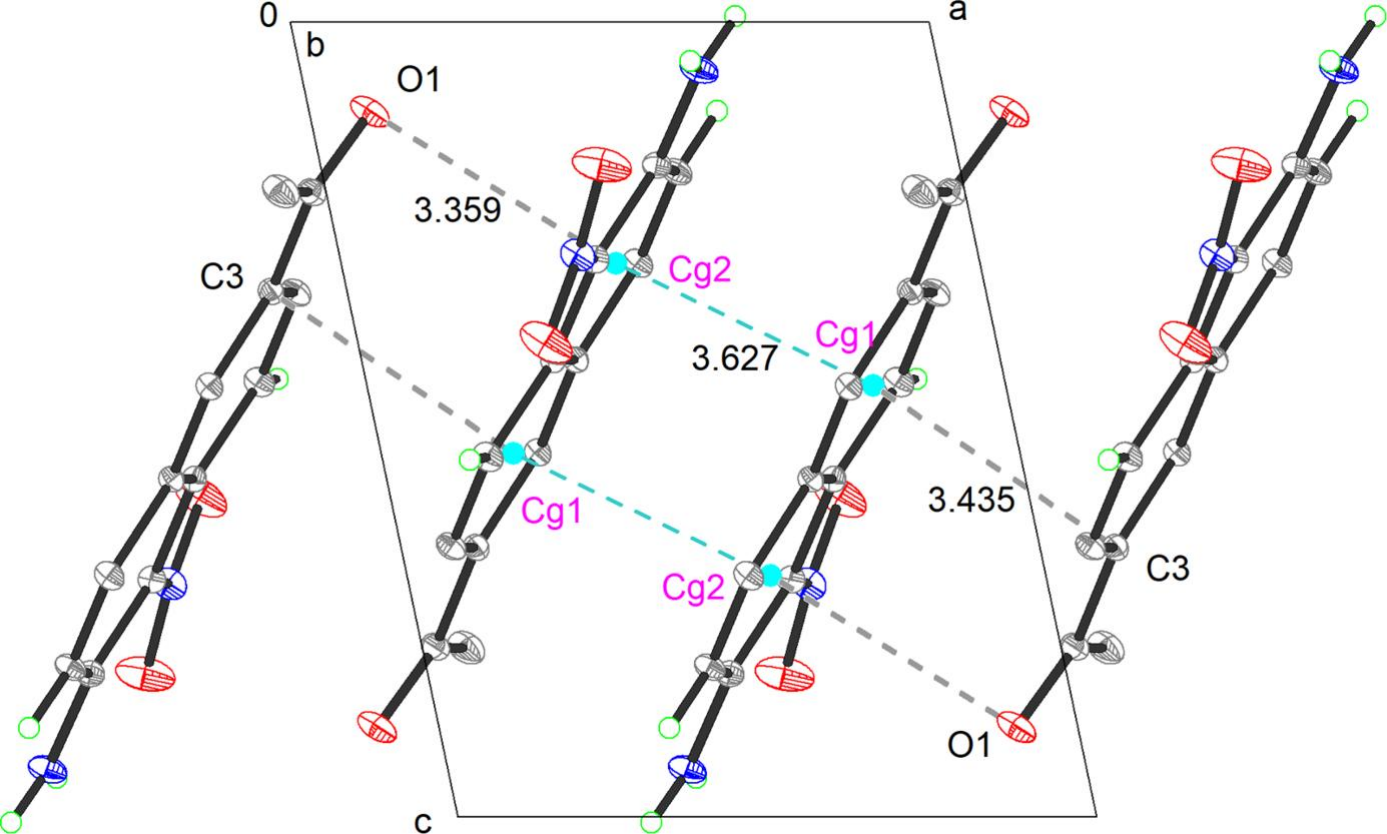
The packing of the mol­ecules showing the *n*–π and π–π stacking inter­actions (dashed lines) along the *a*-axis direction.

The title compound (Fig. 1 ▸) comprises a naphthalene core structure, where all carbon atoms within the naphthalene ring system (C1–C10) are ideally *sp* ^2^-hybridized. The amino group and the nitro group are adjacent, located at positions C-7 and C-8, respectively, of the naphthalene ring system,while the acetyl group is located at the C-3 position. The angles between the two hydrogen atoms on the amino group and between the two oxygen atoms on the nitro group are 120 and 118.66 (17)°, respectively. The O2—N1—C8—C9 and O2—N1—C8—C7 torsion angles are 112.80 (3) and −165.8 (2)°, respectively. The acetyl group and naphthalene ring system are almost coplanar, the O1—C11—C3—C2 and C12—C11—C3—C4 torsion angles being 2.00 (3) and 2.80 (3)°, respectively. The intra­molecular N2—H2*A*⋯O and C1—H1⋯O2 hydrogen bonds (Table 1 ▸) lead to the formation of two six-membered rings, stabilizing the mol­ecular conformation (Fig. 1 ▸). The structure of **I** is further stabilized by atom–centroid and centroid–centroid (*Cg*–*Cg*) inter­actions, illustrated in Fig. 2 ▸.

## 3. Supra­molecular features

**Figure 3:**
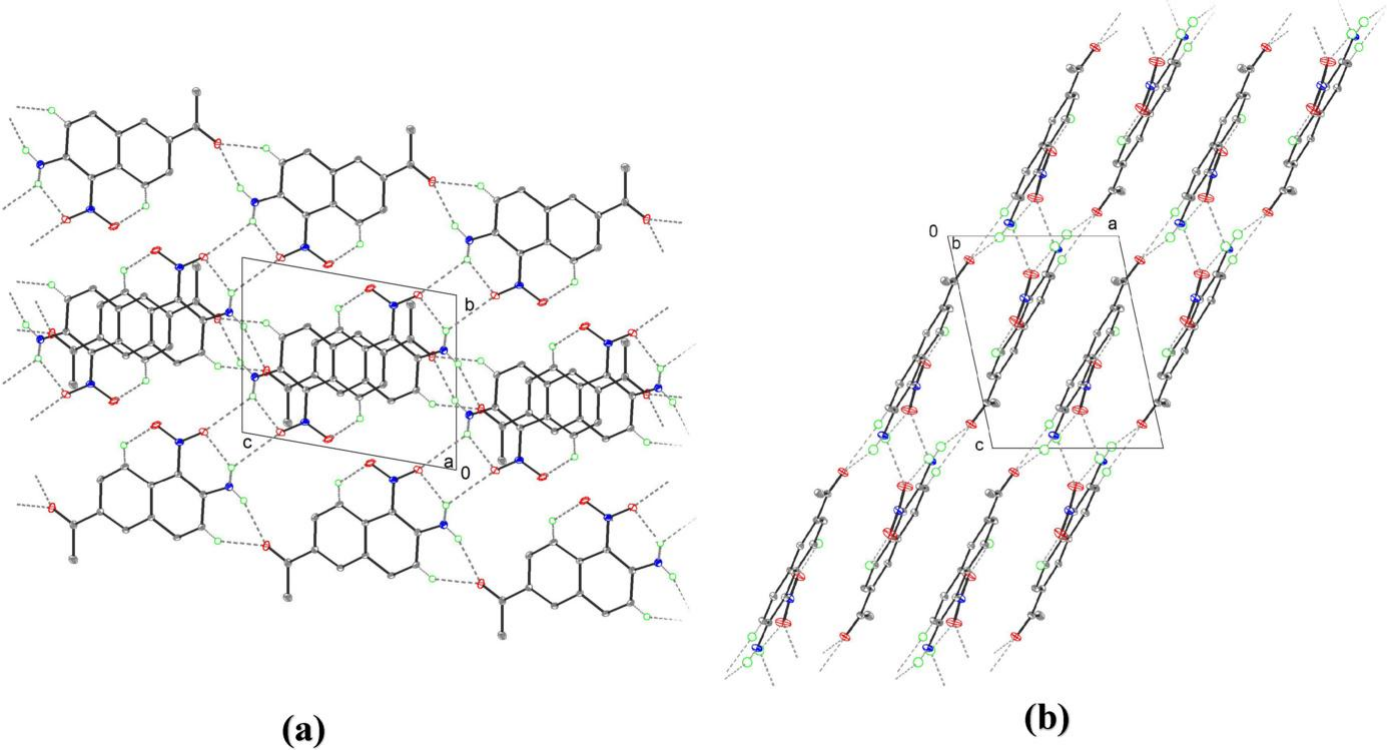
The packing of mol­ecules showing the hydrogen-bonding inter­actions (gray dashed lines) along (*a*) the *a*-axis direction and (*b*) the *b*-axis direction.

In the crystal, the mol­ecules are linked *via* C6—H6⋯O1 and N2—H2*B*⋯O1 hydrogen bonds (Table 1 ▸), generating two-dimensional layers propagating along the [101] direction (Fig. 3 ▸). Two-dimensional layers formed by N2—H2*A*⋯O3 inter­molecular hydrogen bonds (Fig. 3 ▸) while *n*–π and π–π stacking inter­action form a super three-dimensional network structure (Fig. 2 ▸). The π–π inter­actions are medium-to-weak (*Cg*1–*Cg*2 distances greater than 3 Å with a slippage value 3.627 Å where *Cg*1 and *Cg*2 are the centroids of the C1–C4/C10/C9 and C5–C10 rings, respectively). In addition, The structure exhibits typical *n*–π (O1⋯*Cg*2 = 3.359 Å) and van der Waals interactions (C3⋯*Cg*1 = 3.435  Å).

## 4. Database survey

A survey of the Cambridge Structural Database (CSD version 2024.1.0; Groom *et al.*, 2016 ▸) revealed a total of nine compounds with structural similarity greater than 70%, of which six have an acetyl or nitro substituent connected to the naphthalene ring core structure. However, there is only one compound with both acetyl and amino groups on the naphthalene ring system (refcode EBUXIL, CCDC 955350; Rejc *et al.*, 2014 ▸).

## 5. Hirshfeld Surface analysis

**Figure 4:**
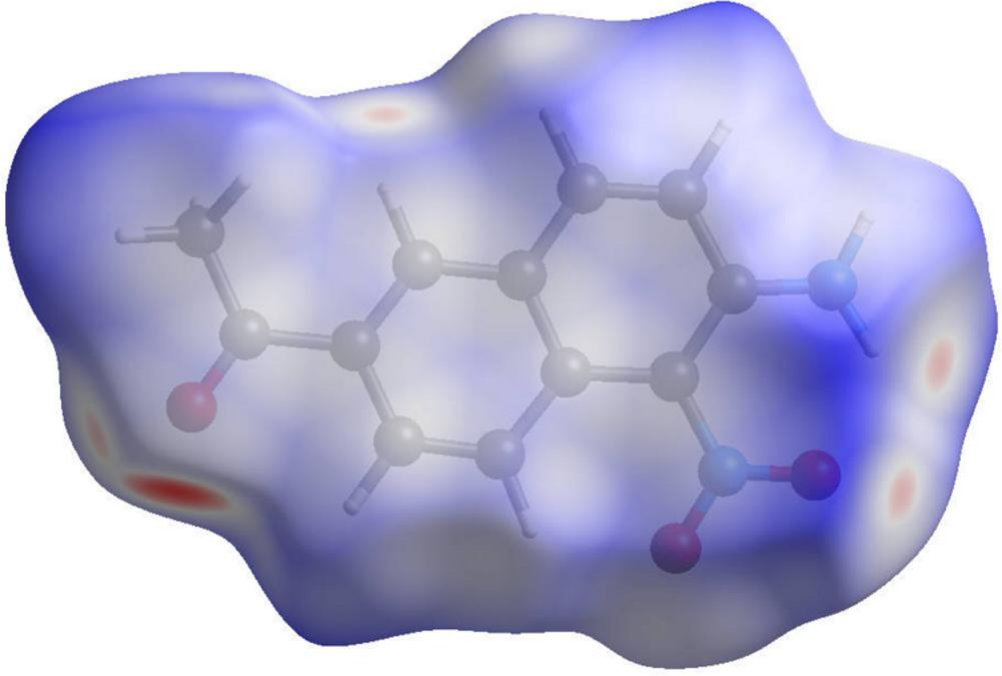
View of the three-dimensional Hirshfeld surface mapped over *d*_norm_.

**Figure 5:**
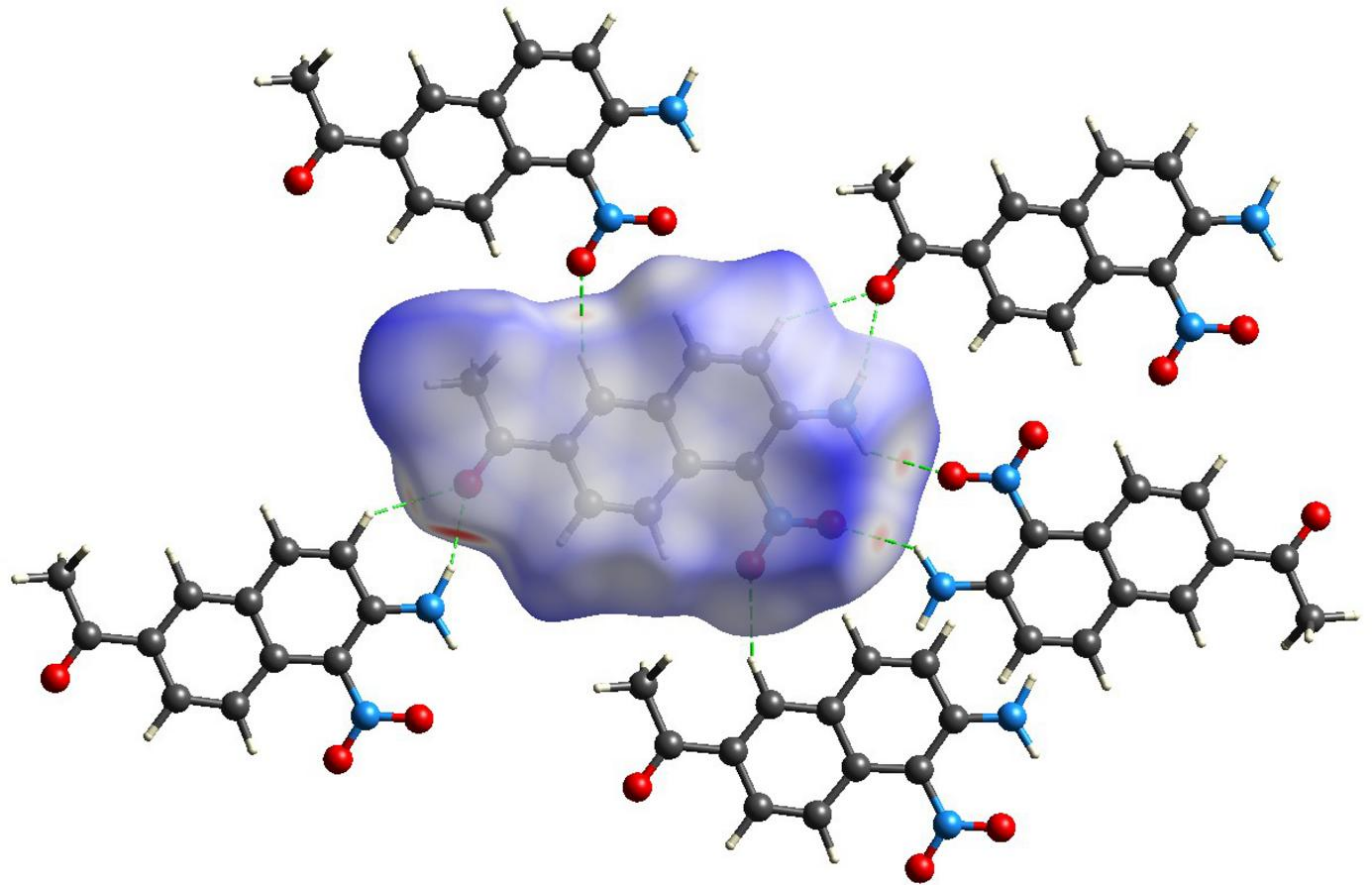
Hirshfeld surface mapped over *d*_norm_ showing H⋯O/O⋯H, H⋯N/N⋯H, and C⋯H/H⋯C contacts.

**Figure 6:**
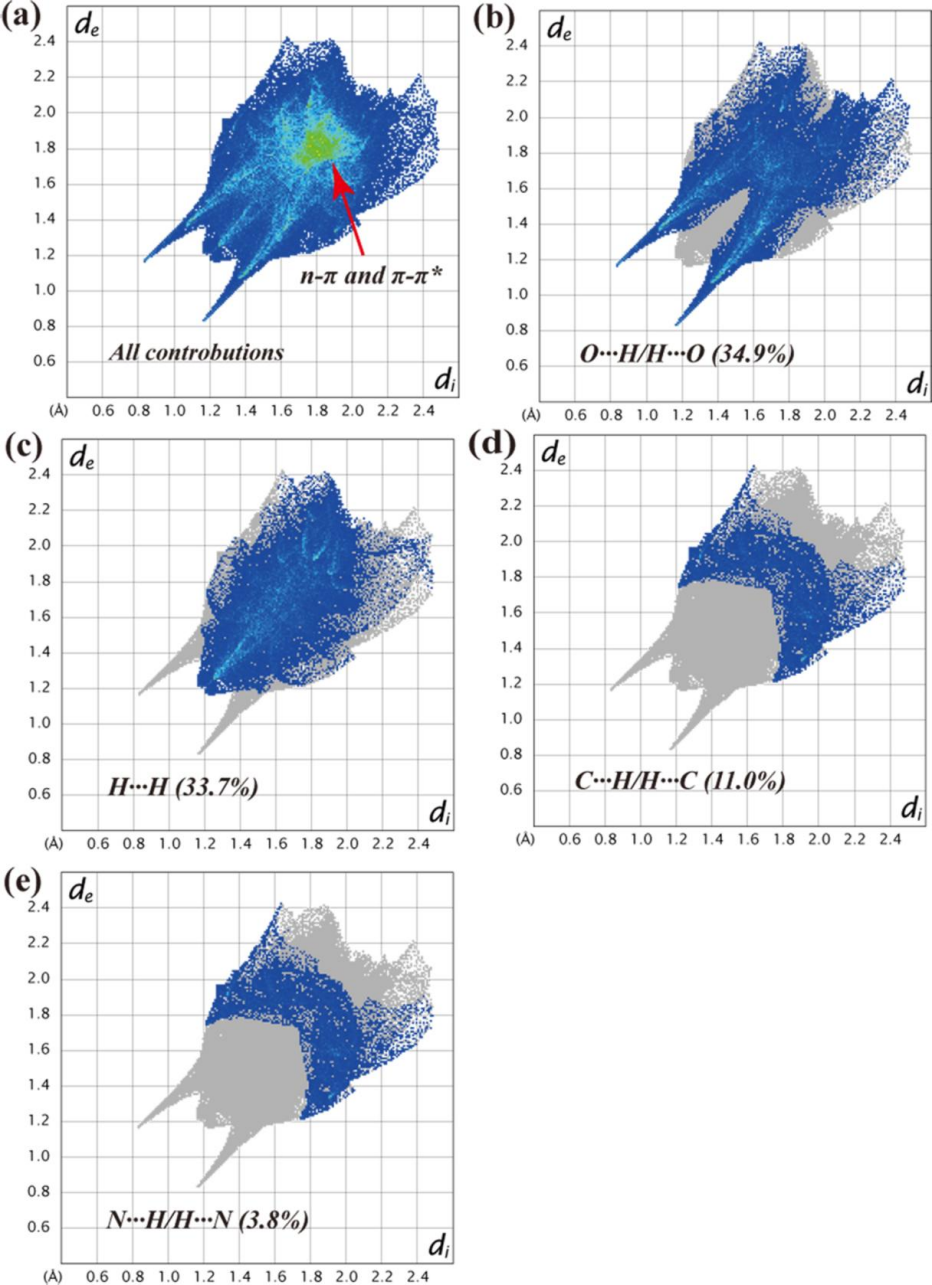
The two-dimensional fingerprint plots showing (*a*) all inter­actions, and delineated into (*b*) O⋯H/H⋯O, (*c*) H⋯H, (*d*) C⋯H/H⋯C, and (*f*) N⋯H/H⋯N inter­actions [the *d*_e_ and *d*_i_ values represent the distances (in Å) from a point on the Hirshfeld surface to the nearest atoms inside and outside the surface, respectively].

**Figure 7:**
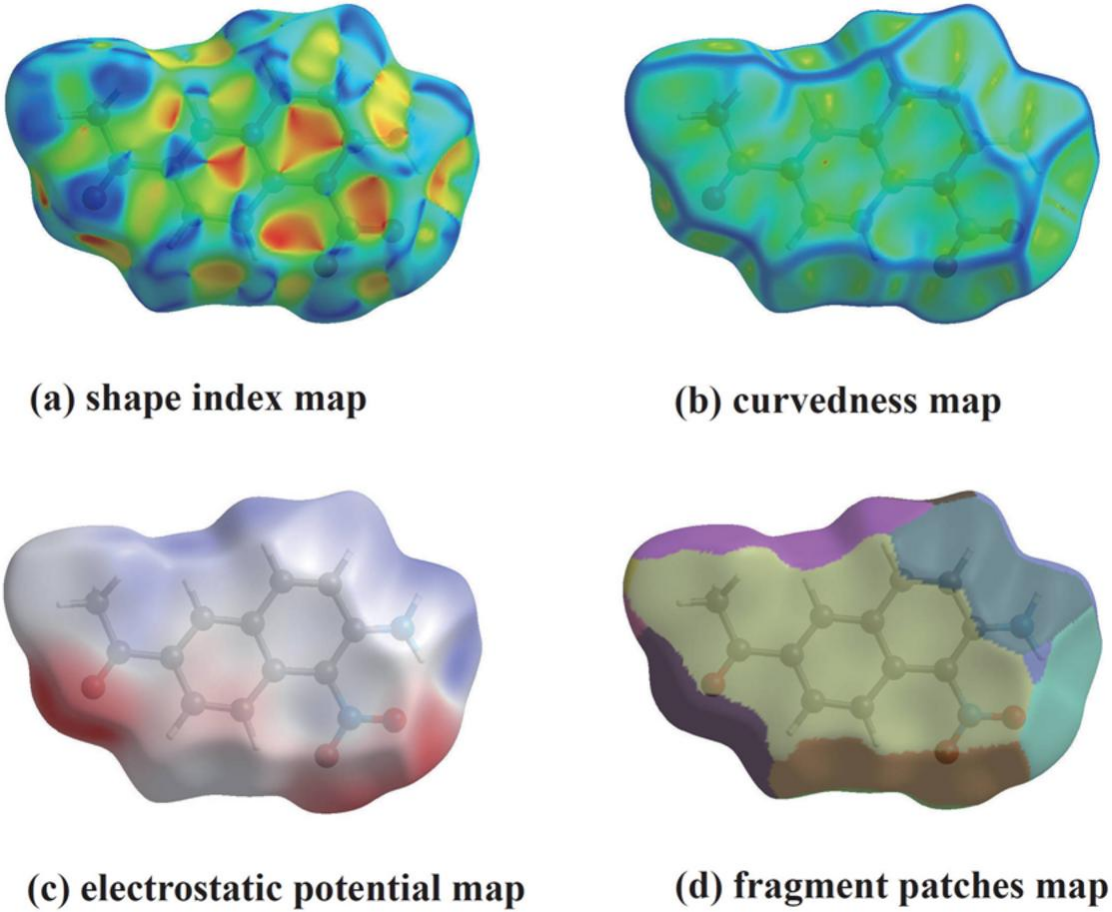
Hirshfeld surfaces mapped over (*a*) electrostatic potential, (*b*) shape-index, (*c*) curvedness, and (*d*) fragment patches.

In order to visualize the inter­molecular inter­actions, a Hirshfeld surface analysis (Hirshfeld, 1977 ▸) was carried out using *Crystal Explorer 21.5* (Spackman *et al.*, 2021 ▸). The three-dimensional *d*_norm_ surface of the title compound, plotted with a standardized resolution and color scale ranging from −0.4536 (red) to 1.4893 (blue) a.u. is shown in Fig. 4 ▸. It reveals the primary inter­actions to be inter­nal and external hydrogen bonds, *n*–π and π–π inter­actions. The intense red spots symbolize short contacts and negative *d*_norm_ values on the surface are related to the presence of the N2—H2*A*⋯O3 hydrogen bonds in the crystal structure. Weak C1—H1⋯O2 and C6—H6⋯O1 contacts are showed by dim red spots (Fig. 5 ▸). The 2D fingerprint plots qu­anti­tatively visualize the H⋯O/O⋯H, H⋯H, H⋯C/C⋯H, and H⋯N/N⋯H inter­actions (Fig. 6 ▸). The *n*–π and π–π stacking inter­actions, located in the middle region of the fingerprint plot, play an integral role in the overall crystal packing, contributing 16.6% (Fig. 6 ▸ *a*). The most significant contacts are H⋯O/O⋯H and H⋯H, contributing 34.9% and 33.7%, respectively, while the H⋯C/C⋯H contacts contribute 11.0%, and the H⋯N/N⋯H contacts contribute 3.8% to the Hirshfeld surface (Fig. 6 ▸ *b*–6*e*). The Hirshfeld surfaces mapped over shape-index, curvedness, electrostatic potential, and fragment patches are shown in Fig. 7 ▸. The pattern of orange and blue triangles on the shape-index surface (Fig. 7 ▸ *a*) shows the characteristic feature of π–π inter­actions. Since curvedness plot (Fig. 7 ▸ *b*) shows flat regions, it is evident that the title mol­ecules are arranged in planar stacking (Spackman *et al.*, 2009 ▸).

## 6. DFT calculations

The mol­ecular structure of the title compound in the gas phase was optimized using density functional theory (DFT) (Neese *et al.*, 2009 ▸) with the standard B3LYP-D3BJ method with the basis set def2-TZVP (Hanwell *et al.*, 2012 ▸), default SCF and geometrical convergence criteria as implemented in the *Orca 5.0.4* package (Neese, 2018 ▸, 2022 ▸). The input files were prepared from the CIF file using *Avogadro 1.98.1* software (Hanwell *et al.*, 2012 ▸). The calculated bond lengths and bond angles for the title compound are presented in Table 2 ▸ along with the corresponding crystallographic data (from the CIF file) for comparison·The computed results agree well with the experimental crystallographic data.

**Figure 8:**
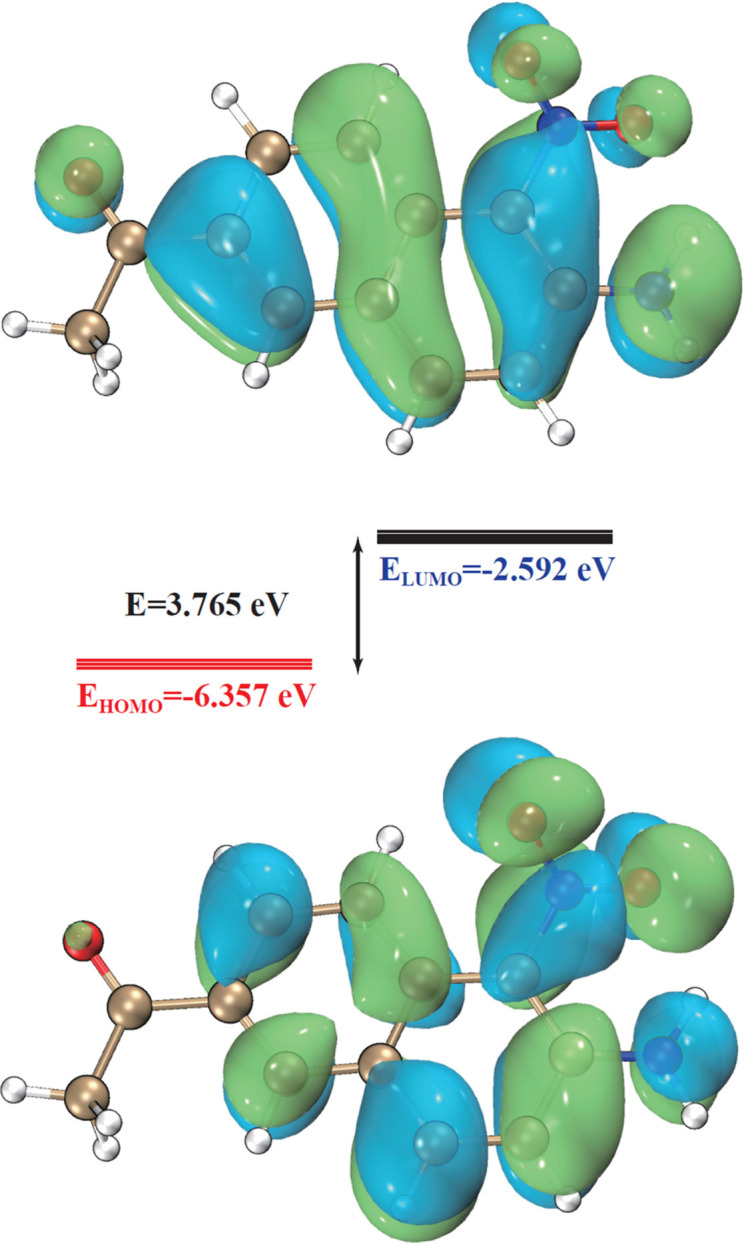
HOMO and LUMO calculated by the B3LYP-D3BJ/def2-TZVP method. The energy band gap is shown.

Electron distribution in the frontier mol­ecular orbital (FMOs), *i.e.* the highest occupied MO (HOMO; −6.357 eV) and the lowest unoccupied MO (LUMO; −2.592 eV) with a LUMO–HOMO gap of 3.765 eV, are illustrated in Fig. 8 ▸. The HOMO is less distributed on the naphthyl acetyl group while LUMO is more distributed. When the energy gap is small, the mol­ecule exhibits high polarizability and enhances its chemical reactivity. The calculated energies and related parameters are presented in Table 3 ▸. The hardness and softness values are important parameters in understanding the chemical reactivity of a compound and stability index of a ligand. Compounds formed with a ligand exhibiting higher dipole moment values are generally more stable (Zhan *et al.*, 2003 ▸).

## 7. Mol­ecular electrostatic potential (MEP)

**Figure 9:**
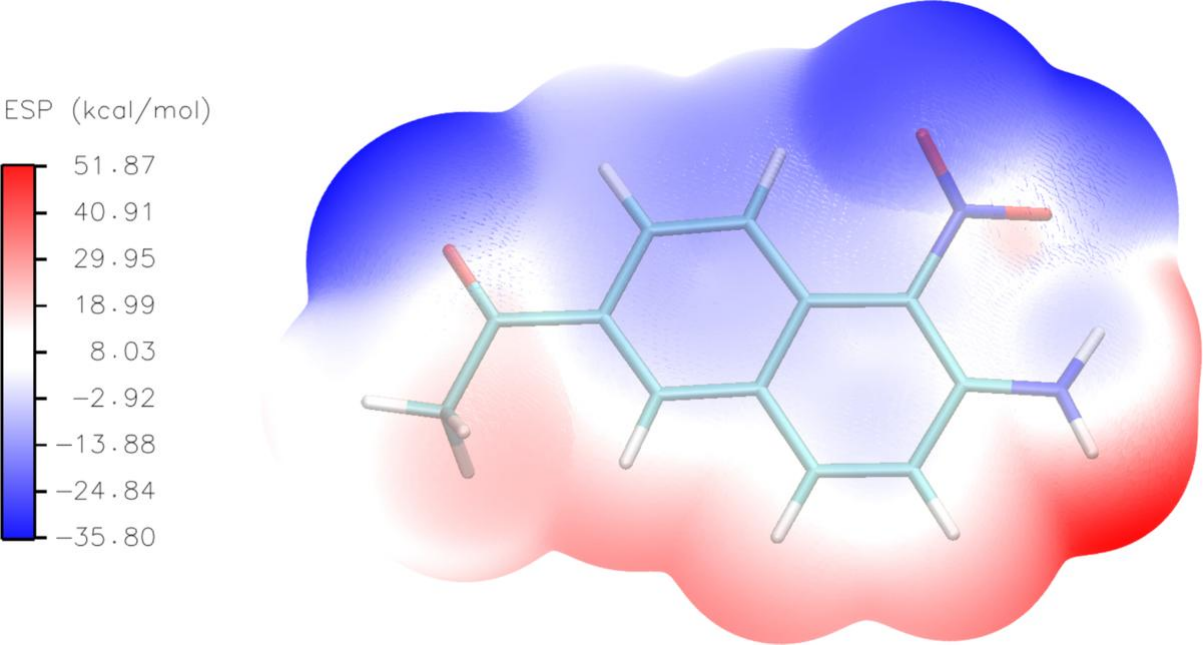
Mol­ecular electrostatic potential (MEP) surfaces mapped from the optimized geometries of the ωB97*M*-V/def2-TZVP calculation.

The mol­ecular electrostatic potential (MEP) map, generated using ωB97M-V/def2-TZVP (Mardirossian & Head-Gordon, 2016 ▸) basis sets with the *Orca 5.0.4* software package (Neese, 2022 ▸), was used to broadly predict reactive sites for electrophilic and nucleophilic attack in the title compound. The map, drawn using *VMD 1.9.4* (Humphrey & Schulten, 1996 ▸) and *Multiwfn 3.8* (Lu & Chen, 2012 ▸; Zhang & Lu, 2021 ▸), is shown in Fig. 9 ▸. In the crystal, the mol­ecular charge distribution is governed by the MEP. The electrostatic potential in the MEP map varies increasingly according to a red < white < blue color scheme [ranging from −35.80 kcal mol^−1^ (extreme red) to 51.87 kcal mol^−1^ (extreme blue)].

## 8. Synthesis and crystallization

0.5 g of 2-acetyl-6-acetamido-5-nitro­naphthalene were dissolved in 30 mL of MeOH, 3 mL of concentrated H_2_SO_4_ was, and the reaction was refluxed at 353 K for 6 h. After the reaction was complete, it was quenched with 10 mL of ice water, precipitating yellow solids, and filtered to obtain the target product. The MeOH was dissolved and red transparent block-shaped crystals were cultured at 277 K in the refrigerator (Xu *et al.*, 2017 ▸).

## 9. Refinement

Crystal data, data collection and structure refinement details are summarized in Table 4 ▸. H atoms were positioned geometrically (C—H = 0.93–0.96 Å and N—H = 0.86 Å) and refined as riding, with *U*_iso_(H) = 1.2*U*_eq_(N) for NH hydrogen atoms or 1.5*U*_eq_(C-meth­yl).

## Supplementary Material

Crystal structure: contains datablock(s) I. DOI: 10.1107/S2056989024003797/zn2036sup1.cif

Supporting information file. DOI: 10.1107/S2056989024003797/zn2036Isup3.cml

Structure factors: contains datablock(s) I. DOI: 10.1107/S2056989024003797/zn2036Isup3.hkl

CCDC reference: 2350991

Additional supporting information:  crystallographic information; 3D view; checkCIF report

## Figures and Tables

**Table 1:** Hydrogen-bond geometry (Å, °)

*D*—H⋯*A*	*D*—H	H⋯*A*	*D*⋯*A*	*D*—H⋯*A*
N2—H2*B*⋯O1^i^	0.86	2.16	2.988 (2)	162
N2—H2*A*⋯O3^ii^	0.86	2.54	3.146 (3)	128
N2—H2*A*⋯O3	0.86	1.95	2.556 (3)	127
C6—H6⋯O1^i^	0.93	2.57	3.341 (2)	141
C1—H1⋯O2	0.93	2.11	2.720 (3)	122

Symmetry codes: (i) 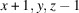 ; (ii) 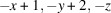 .

**Table 2:** Comparison of selected (X-ray and DFT) geometric data (Å, °)

Bonds/angles	X-ray	ωB97*M*-V/def2-TZVP
C12—C11	1.492 (3)	1.514
C10—C4	1.398 (2)	1.406
C11—C3	1.483 (3)	1.491
C10—C5	1.422 (2)	1.419
C11—O1	1.215 (2)	1.215
C5—C6	1.338 (3)	1.354
C3—C2	1.408 (3)	1.410
C6—C7	1.431 (3)	1.427
C3—C4	1.373 (2)	1.378
C7—C8	1.412 (3)	1.410
C2—C1	1.366 (3)	1.371
C7—N2	1.333 (2)	1.348
C1—C9	1.416 (3)	1.418
C8—N1	1.425 (2)	1.446
C9—C10	1.424 (2)	1.427
N1—O2	1.217 (2)	1.223
C9—C8	1.447 (3)	1.437
N1—O3	1.227 (2)	1.240
C3—C11—C12	119.48 (18)	118.71
C5—C10—C9	119.73 (16)	119.45
O1—C11—C12	119.56 (19)	120.66
C3—C4—C10	122.33 (17)	121.48
O1—C11—C3	120.96 (19)	120.63
C6—C5—C10	122.09 (17)	121.61
C2—C3—C11	120.46 (17)	118.95
C5—C6—C7	121.51 (17)	119.00
C4—C3—C11	122.46 (17)	122.97
C8—C7—C6	117.75 (16)	117.98
C4—C3—C2	117.08 (17)	118.02
N2—C7—C6	115.85 (17)	117.13
C1—C2—C3	122.07 (17)	121.89
N2—C7—C8	126.40 (17)	124.87
C2—C1—C9	121.76 (17)	121.22
C7—C8—C9	121.77 (16)	121.48
C1—C9—C10	116.07 (16)	116.84
C7—C8—N1	118.03 (16)	118.18
C1—C9—C8	126.75 (16)	125.24
N1—C8—C9	120.18 (16)	120.31
C10—C9—C8	117.15 (16)	117.85
O2—N1—C8	120.96 (17)	119.41
C4—C10—C9	120.65 (16)	120.53
O2—N1—O3	118.66 (17)	121.99
C4—C10—C5	119.62 (16)	120.01
O3—N1—C8	120.21 (18)	118.58

**Table 3:** Calculated energies for the title compound

Mol­ecular energy	Compound (I)
Total energy, *TE* (eV)	−21726.75
*E*_HOMO_ (eV)	− 6.357
*E*_LUMO_ (eV)	−2.592
Gap, Δ*E*(eV)	3.765
Dipole moment, *μ* (Debye)	7.33
Ionization potential, *I* (eV)	8.16
Electron affinity, *A*	0.77
Electronegativity, *χ*	4.46
Hardness,*η*	7.40
Electrophilicity index, *ω*	1.34
Softness, *σ*	0.14
Fraction of electron transferred, Δ*N*	0.69

**Table 4:** Experimental details

Crystal data	
Chemical formula	C_12_H_10_N_2_O_3_
*M*_r_	230.22
Crystal system, space group	Triclinic, *P* 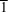
Temperature (K)	296
*a*, *b*, *c* (Å)	8.1208 (13), 8.2262 (14), 9.5944 (15)
α, β, γ (°)	73.338 (4), 72.167 (4), 62.966 (4)
*V* (Å^3^)	535.19 (15)
*Z*	2
Radiation type	Mo *K*α
μ (mm^−1^)	0.11
Crystal size (mm)	0.40 × 0.30 × 0.15

Data collection	
Diffractometer	Bruker *SMART* CCD
Absorption correction	Multi-scan *SADABS*; Krause *et al.*, 2015 ▸)
No. of measured, independent and observed [*I* > 2σ(*I*)] reflections	3406, 1884, 1448
*R*_int_	0.013
(sin θ/λ)_max_ (Å^−1^)	0.594

Refinement	
*R*[*F* ^2^ > 2σ(*F* ^2^)], *wR*(*F* ^2^), *S*	0.051, 0.148, 1.03
No. of reflections	1884
No. of parameters	155
H-atom treatment	H-atom parameters constrained
Δρ_max_, Δρ_min_ (e Å^−3^)	0.41, −0.30

Computer programs: *SMART* and *SAINT* (Bruker, 2002 ▸), *SHELXT2014/7* (Sheldrick, 2015*a* ▸), *SHELXL2014/7* (Sheldrick, 2015*b* ▸) and *publCIF* (Westrip, 2010 ▸).

## supplementary crystallographic information

### Crystal data

**Table:**

C_12_H_10_N_2_O_3_	*Z* = 2
*M**_r_* = 230.22	*F*(000) = 240
Triclinic, *P*1	*D*_x_ = 1.429 Mg m^−^^3^
*a* = 8.1208 (13) Å	Mo *K*α radiation, λ = 0.71073 Å
*b* = 8.2262 (14) Å	Cell parameters from 1265 reflections
*c* = 9.5944 (15) Å	θ = 2.8–25.0°
α = 73.338 (4)°	µ = 0.11 mm^−^^1^
β = 72.167 (4)°	*T* = 296 K
γ = 62.966 (4)°	Block, red
*V* = 535.19 (15) Å^3^	0.40 × 0.30 × 0.15 mm

### Data collection

**Table:**

Bruker SMART CCD diffractometer	1448 reflections with *I* > 2σ(*I*)
phi and ω scans	*R*_int_ = 0.013
Absorption correction: multi-scan SADABS; Krause *et al.*, 2015)	θ_max_ = 25.0°, θ_min_ = 2.3°
	*h* = −9→9
3406 measured reflections	*k* = −6→9
1884 independent reflections	*l* = −11→11

### Refinement

**Table:**

Refinement on *F*^2^	0 restraints
Least-squares matrix: full	Hydrogen site location: inferred from neighbouring sites
*R*[*F*^2^ > 2σ(*F*^2^)] = 0.051	H-atom parameters constrained
*wR*(*F*^2^) = 0.148	*w* = 1/[σ^2^(*F*_o_^2^) + (0.0735*P*)^2^ + 0.2098*P*] where *P* = (*F*_o_^2^ + 2*F*_c_^2^)/3
*S* = 1.03	(Δ/σ)_max_ < 0.001
1884 reflections	Δρ_max_ = 0.41 e Å^−^^3^
155 parameters	Δρ_min_ = −0.30 e Å^−^^3^

### Special details

**Table:**

Geometry. All esds (except the esd in the dihedral angle between two l.s. planes) are estimated using the full covariance matrix. The cell esds are taken into account individually in the estimation of esds in distances, angles and torsion angles; correlations between esds in cell parameters are only used when they are defined by crystal symmetry. An approximate (isotropic) treatment of cell esds is used for estimating esds involving l.s. planes.

### Fractional atomic coordinates and isotropic or equivalent isotropic displacement parameters (Å^2^)

**Table:**

	*x*	*y*	*z*	*U*_iso_*/*U*_eq_	
C12	0.0695 (3)	0.0991 (3)	0.7872 (3)	0.0632 (7)	
H12A	0.1914	0.0236	0.8117	0.095*	
H12B	0.0717	0.0847	0.6908	0.095*	
H12C	−0.0238	0.0615	0.8599	0.095*	
C11	0.0216 (3)	0.2974 (3)	0.7860 (2)	0.0456 (5)	
C3	0.1180 (3)	0.3996 (3)	0.6612 (2)	0.0372 (4)	
C2	0.0793 (3)	0.5851 (3)	0.6600 (2)	0.0444 (5)	
H2	−0.0081	0.6426	0.7383	0.053*	
C1	0.1661 (3)	0.6831 (3)	0.5474 (2)	0.0423 (5)	
H1	0.1366	0.8052	0.5514	0.051*	
C9	0.2999 (2)	0.6039 (2)	0.4247 (2)	0.0324 (4)	
C10	0.3356 (2)	0.4177 (2)	0.42440 (19)	0.0328 (4)	
C4	0.2454 (3)	0.3209 (2)	0.5420 (2)	0.0357 (4)	
H4	0.2726	0.1990	0.5396	0.043*	
C5	0.4643 (3)	0.3292 (2)	0.3033 (2)	0.0392 (5)	
H5	0.4859	0.2079	0.3040	0.047*	
C6	0.5553 (3)	0.4150 (3)	0.1883 (2)	0.0408 (5)	
H6	0.6395	0.3514	0.1120	0.049*	
C7	0.5262 (3)	0.6024 (3)	0.1800 (2)	0.0380 (5)	
C8	0.3990 (3)	0.6942 (2)	0.2985 (2)	0.0357 (4)	
N1	0.3732 (3)	0.8791 (2)	0.2929 (2)	0.0489 (5)	
N2	0.6247 (3)	0.6726 (3)	0.05977 (18)	0.0538 (5)	
H2A	0.6136	0.7843	0.0491	0.065*	
H2B	0.6992	0.6062	−0.0071	0.065*	
O1	−0.0958 (2)	0.3728 (2)	0.88712 (17)	0.0677 (5)	
O2	0.2953 (3)	0.9525 (2)	0.4026 (2)	0.0887 (7)	
O3	0.4405 (4)	0.9628 (2)	0.1795 (2)	0.0975 (8)	

### Atomic displacement parameters (Å^2^)

**Table:**

	*U*^11^	*U*^22^	*U*^33^	*U*^12^	*U*^13^	*U*^23^
C12	0.0646 (15)	0.0471 (13)	0.0580 (14)	−0.0232 (12)	0.0059 (11)	0.0006 (11)
C11	0.0401 (11)	0.0489 (12)	0.0382 (10)	−0.0153 (9)	−0.0010 (9)	−0.0056 (9)
C3	0.0336 (9)	0.0373 (10)	0.0363 (10)	−0.0123 (8)	−0.0038 (8)	−0.0075 (8)
C2	0.0427 (11)	0.0456 (11)	0.0397 (10)	−0.0137 (9)	0.0033 (8)	−0.0196 (9)
C1	0.0473 (11)	0.0309 (10)	0.0467 (11)	−0.0133 (9)	−0.0025 (9)	−0.0156 (8)
C9	0.0331 (9)	0.0280 (9)	0.0359 (10)	−0.0104 (7)	−0.0072 (8)	−0.0086 (7)
C10	0.0334 (9)	0.0293 (9)	0.0357 (9)	−0.0120 (8)	−0.0039 (7)	−0.0100 (7)
C4	0.0366 (10)	0.0301 (9)	0.0389 (10)	−0.0137 (8)	−0.0037 (8)	−0.0078 (8)
C5	0.0437 (11)	0.0283 (9)	0.0437 (11)	−0.0145 (8)	0.0003 (8)	−0.0135 (8)
C6	0.0449 (11)	0.0371 (10)	0.0372 (10)	−0.0160 (9)	0.0036 (8)	−0.0154 (8)
C7	0.0427 (11)	0.0361 (10)	0.0357 (10)	−0.0186 (9)	−0.0058 (8)	−0.0049 (8)
C8	0.0409 (10)	0.0271 (9)	0.0399 (10)	−0.0139 (8)	−0.0078 (8)	−0.0072 (8)
N1	0.0591 (11)	0.0313 (9)	0.0543 (11)	−0.0213 (8)	−0.0023 (8)	−0.0092 (8)
N2	0.0723 (13)	0.0476 (10)	0.0405 (10)	−0.0344 (10)	0.0068 (9)	−0.0083 (8)
O1	0.0671 (11)	0.0683 (11)	0.0509 (9)	−0.0280 (9)	0.0205 (8)	−0.0200 (8)
O2	0.1203 (16)	0.0512 (10)	0.0912 (14)	−0.0504 (11)	0.0344 (12)	−0.0407 (10)
O3	0.174 (2)	0.0506 (11)	0.0627 (11)	−0.0669 (13)	0.0141 (12)	−0.0071 (9)

### Geometric parameters (Å, º)

**Table:**

C12—C11	1.492 (3)	C10—C4	1.398 (2)
C11—O1	1.215 (2)	C10—C5	1.422 (2)
C11—C3	1.483 (3)	C5—C6	1.338 (3)
C3—C4	1.373 (2)	C6—C7	1.431 (3)
C3—C2	1.408 (3)	C7—N2	1.333 (2)
C2—C1	1.366 (3)	C7—C8	1.412 (3)
C1—C9	1.416 (3)	C8—N1	1.425 (2)
C9—C10	1.424 (2)	N1—O2	1.217 (2)
C9—C8	1.447 (3)	N1—O3	1.227 (2)
			
O1—C11—C3	120.96 (19)	C5—C10—C9	119.73 (16)
O1—C11—C12	119.56 (19)	C3—C4—C10	122.33 (17)
C3—C11—C12	119.48 (18)	C6—C5—C10	122.09 (17)
C4—C3—C2	117.08 (17)	C5—C6—C7	121.51 (17)
C4—C3—C11	122.46 (17)	N2—C7—C8	126.40 (17)
C2—C3—C11	120.46 (17)	N2—C7—C6	115.85 (17)
C1—C2—C3	122.07 (17)	C8—C7—C6	117.75 (16)
C2—C1—C9	121.76 (17)	C7—C8—N1	118.03 (16)
C1—C9—C10	116.07 (16)	C7—C8—C9	121.77 (16)
C1—C9—C8	126.75 (16)	N1—C8—C9	120.18 (16)
C10—C9—C8	117.15 (16)	O2—N1—O3	118.66 (17)
C4—C10—C5	119.62 (16)	O2—N1—C8	120.96 (17)
C4—C10—C9	120.65 (16)	O3—N1—C8	120.21 (18)
			
O1—C11—C3—C4	−177.21 (19)	C4—C10—C5—C6	−179.61 (18)
C12—C11—C3—C4	2.8 (3)	C9—C10—C5—C6	0.4 (3)
O1—C11—C3—C2	2.0 (3)	C10—C5—C6—C7	−0.8 (3)
C12—C11—C3—C2	−178.01 (19)	C5—C6—C7—N2	−179.82 (18)
C4—C3—C2—C1	−1.1 (3)	C5—C6—C7—C8	0.6 (3)
C11—C3—C2—C1	179.64 (18)	N2—C7—C8—N1	−1.2 (3)
C3—C2—C1—C9	0.2 (3)	C6—C7—C8—N1	178.35 (17)
C2—C1—C9—C10	1.1 (3)	N2—C7—C8—C9	−179.66 (18)
C2—C1—C9—C8	179.23 (18)	C6—C7—C8—C9	−0.2 (3)
C1—C9—C10—C4	−1.7 (3)	C1—C9—C8—C7	−178.23 (18)
C8—C9—C10—C4	−179.93 (16)	C10—C9—C8—C7	−0.2 (3)
C1—C9—C10—C5	178.33 (16)	C1—C9—C8—N1	3.3 (3)
C8—C9—C10—C5	0.1 (3)	C10—C9—C8—N1	−178.64 (16)
C2—C3—C4—C10	0.6 (3)	C7—C8—N1—O2	−165.8 (2)
C11—C3—C4—C10	179.81 (17)	C9—C8—N1—O2	12.8 (3)
C5—C10—C4—C3	−179.17 (17)	C7—C8—N1—O3	9.4 (3)
C9—C10—C4—C3	0.8 (3)	C9—C8—N1—O3	−172.1 (2)

### Hydrogen-bond geometry (Å, º)

**Table:**

*D*—H···*A*	*D*—H	H···*A*	*D*···*A*	*D*—H···*A*
N2—H2*B*···O1^i^	0.86	2.16	2.988 (2)	162
N2—H2*A*···O3^ii^	0.86	2.54	3.146 (3)	128
N2—H2*A*···O3	0.86	1.95	2.556 (3)	127
C6—H6···O1^i^	0.93	2.57	3.341 (2)	141
C1—H1···O2	0.93	2.11	2.720 (3)	122

Symmetry codes: (i) *x*+1, *y*, *z*−1; (ii) −*x*+1, −*y*+2, −*z*.
